# Hepatic Angiosarcoma Presenting as an Acute Intraabdominal Hemorrhage Treated with Transarterial Chemoembolization

**DOI:** 10.1155/2007/90169

**Published:** 2007-12-03

**Authors:** Glenn William Stambo, Michael J. Guiney

**Affiliations:** ^1^Division of Vascular and Interventional Radiology, Department of Radiology, St. Joseph's Hospital and Medical Center, Tampa, FL 33607, USA; ^2^Department of Radiology, University College London Hospitals NHS Foundation Trust, London NW1 2BU, UK

## Abstract

Primary malignant neoplasms of the liver are some of the most uncommon malignancies in many parts of the world. They include hepatocellular carcinoma and stromal tumors such as hepatic angiosarcoma. It is a lethal tumor with life expectancy of less than six months. Once discovered, it is often too late for surgical intervention. Like other vascular tumors of the liver and spleen, intraperitoneal hemorrhage is a well-documented finding of angiosarcoma which can be lethal if not diagnosed and treated immediately. As in our case, intraperitoneal hemorrhage from primary tumor rupture was the only clinical presentation of this neoplasm. Approximately 15% of patients present with acute hemoperitoneum from either tumor rupture or peritoneal metastasis. Although several therapeutic options are available, we describe apalliative therapy for hepatic angiosarcoma utilizing transcatheter arterial chemoembolization (TACE) techniques incorporating the newer embolization agent Embospheres to locally target and treat this aggressive tumor.

## 1. Report

**Figure 1:**
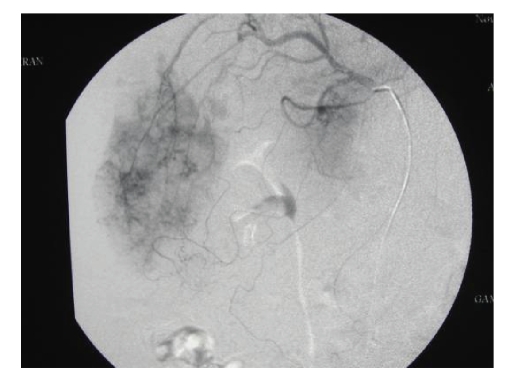
Selective right hepatic arteriogram demonstrating an extensive vascular mass right lobe liver. No evidence of contrast extravasations to suggest acute hemorrhage at this time.

**Figure 2:**
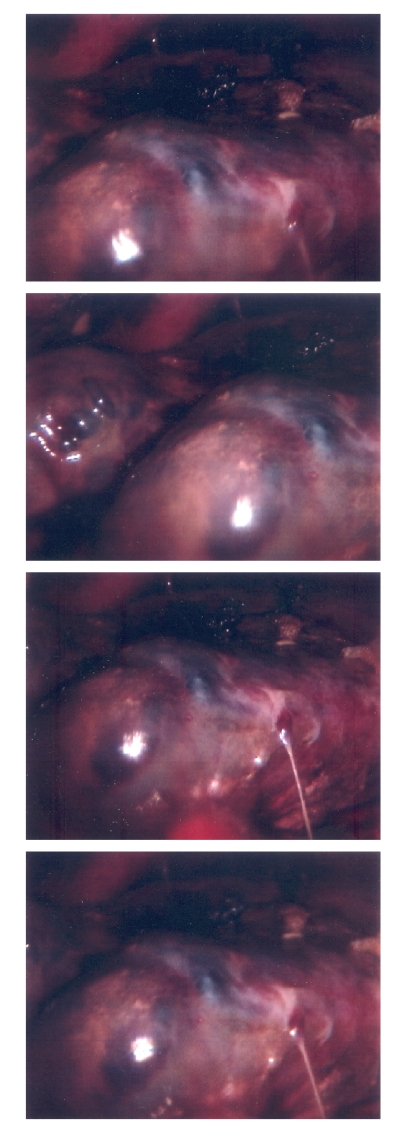
Intraoperative color photograph demonstrating the lobulated blood filled cystic mass consistent with hepatic angiosarcoma.

**Figure 3:**
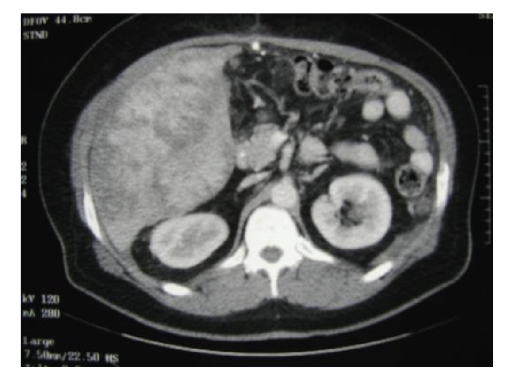
CT scan abdomen with contrast demonstrated highly vascular tumor with heterogeneous uptake pattern consistent with hepatic angiosarcoma.

A 54-year-old male with a past medical history significant for hemachromatosis, coronary bypass graft surgery, with an ejection fraction of 20%, presented to an outside institution with clinical and computed tomography findings of acute intraperitoneal hemorrhage. At that time, an emergent mesenteric angiogram was obtained demonstrating a multicentric pathological vascular mass involving the right hepatic lobe (as in Figure 1). Also, no vascular abnormalities were noted in the left lobe. There was no contrast extravasation identified to suggest vascular rupture and, therefore, no endovascular intervention was performed at the outside hospital. Following the angiogram, a CT-guided percutaneous liver biopsy was performed the next day which was nondiagnostic. Two days following the initial intraperitoneal hemorrhage, the patient was transferred to our tertiary care institution where he was admitted to the surgical service. The patient subsequently underwent an open surgical biopsy the next morning (see Figure 2). The pathologic sample demonstrated a diagnosis of a high-grade hepatic angiosarcoma. Ultrasonography, computed tomography, and magnectic resonance imaging confirmed the findings of a hepatic neoplasm involving a significant portion of the right hepatic lobe (see Figure 3). The portal vein was patent and no extrahepatic disease was present. In light of the patient’s extensive right hepatic involvement and multiple comorbidities, the surgical option was regarded no feasible by the attending hepatic surgeon. The patient was referred to our service for endovascular management. Following consultation with the family discussing the palliative nature of the chemoembolization procedure, the patient was brought to the interventional suite for transarterial chemoembolization (TACE) five days following initial presentation.

**Figure 4:**
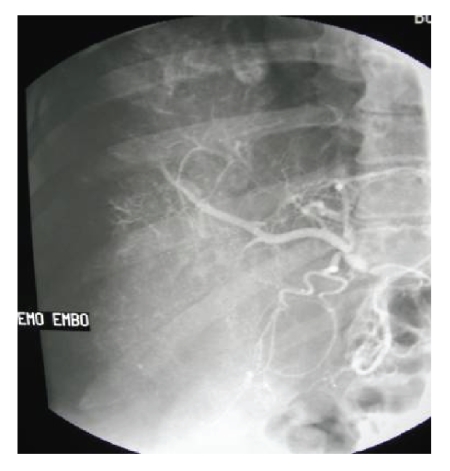
Right hepatic angiogram following chemoembolization of the hepatic angiosarcoma demonstrating the *“*snow storm*”* appearance consistent with diffuse uptake and entrapment of the ethiodol within the tumor.

**Figure 5:**
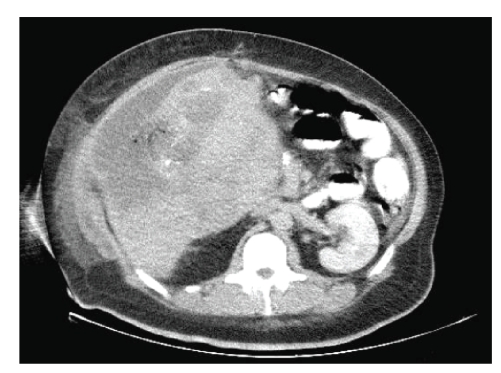
Three month followup CT scan abdomen with contrast demonstrating no enhancing tumor remaining following TACE. Extensive necrosis and deposition of ethiodol trapped within tumor.

A 5 French cobra catheter (Terumo/Boston Scientific, Natick, Mass, USA) was advanced into the right hepatic artery and a selective hepatic arteriogram was obtained with particular attention to pertinent hepatic arterial anatomy. A diffusely vascular enhancing mass consistent with the diagnosis of hepatic angiosarcoma was confirmed along with other satellite masses in the superior surface of the right lobe. Again, no arterial contrast extravasation was noted on this exam to suggest intraperitoneal hemorrhage. The portal vein was patent. TACE was performed with a combination of ethiodol (lipidol) (Savage Laboratories, Melville, NY, USA), Embospheres 500–700 *μ*m (Biosphere Medical, Rockland, Mass, USA) and mixture of Doxorubicin and Mitomycin C (Bedford Laboratories, Bedford, Ohio, USA). During embolization, stasis of flow was identified within the tumor vascularity consistent with occlusion of the neovascular feeding vessels to the mass. Successful embolization was noted fluoroscopically with diffuse uptake of the ethiodol solution within the tumor resulting in a *“*snow storm*”* uptake pattern (see Figure 4). There were no postprocedure complications. The patient was discharged to home in two days without developing postembolization syndrome and was asymptomatic for three months. He was aware that if new lesions or residual enhancing tumor were identified on the followup studies that repeating TACE procedure would be recommended at that time. No further TACE procedures were performed during this time interval and his followup CT imaging at three months demonstrated no residual or new enhancing tumor, diffuse necrosis of the previous tumor bed, and ethiodol scattered throughout the tumor masses (Figure 5). Unfortunately, the patient succumbed to his underlying comorbidities and died suddenly of an acute myocardial infarction four months after his procedure.

## 2. Discussion

Hepatic malignancies include hepatocellular carcinoma, gastrointestinal and nongastrointestinal metastases, and primary or metastatic sarcomas [1]. A primary sarcoma of the liver is the hepatic angiosarcoma. Angiosarcomas account for only 2% of all primary hepatic malignancies [1–6]. They have a high malignant potential resulting in a poor prognosis with death in less than one year. They are surgically unresectable at the time of diagnosis due to multiple tumors, high pathologic grade, and rapid-growing tumor burden [7]. This tumor occurs exclusively in late adulthood to those who have been exposed to environmental toxins such as polyvinylcholoride monomers, thorium dioxide (Thorotrast), and arsenic insecticides [1, 2, 4]. There is a rare correlation between hemachromatosis and hepatic angiosarcoma [1, 3, 7, 8]. Our patient had hemachromatosis without other environmental factors for hepatic angiosarcoma. Furthermore, 15% of angiosarcomas present with intraperitoneal hemorrhage which can be lethal in some patients [2]. Our patient presented with hemoperitoneum from tumor rupture. Furthermore, metastatic liver gastrointestinal stromal tumors are rare but are more common than primary hepatic angiosarcoma. These liver metastases present with bleeding complications (30–40%); and selective arterial embolization has been used for palliation of the gastrointestinal hemorrhage [9]. We describe the first case in the English language of TACE involving an unresectable hepatic angiosarcoma utilizing Embospheres into the neovascular branches of the neoplasm [10, 11]. Due to the large tumor burden, multiple TACE procedures were expected for this patient. However, the response following one TACE with Embospheres was excellent in this short interval. Tumor necrosis and ethiodol material were visualized on followup CT scan images consistent with a good postembolization result. The advantage of TACE is its ability to selectively deliver chemotherapeutic agent and embolic material to the tumor while sparring surrounding liver tissue without concurrent systemic toxicity [12]. We feel that the addition of Embospheres to our TACE regimen has improved tumor killing and subsequent necrosis due to its unique malleable conforming properties not available in other emoblization devices allowing for more homogeneous neovascular arterial occlusion. Embospheres are available in various sizes providing further options for other vascular territories such as uterine artery fibroid embolization.

Recently, transarterial placement of yttrium-90 glass microspheres (90Y-uS; TheraSphere, MDS Nordion, Ottawa, ON, Canada), SIR-sphere (SIRTex Medical Inc., Lake Forest, Ill, USA), and LC beads (Angiodynamics, Queensbury, NY, USA) for the treatment of unresectable hepatocellular carcinoma and colorectal metastasis respectfully are available in the United States [13]. Also, depending on size of the tumor, percutaneous therapy utilizing radio frequency thermal ablation is readily available for various organs [14]. These options may play a role in the palliative management of patients with unresectable angiosarcoma depending on size, location, and tumor burden within the liver. In patients with acceptable liver function, the palliative nature of TACE with its good risk to benefit ratio makes this procedure a useful option with other types of malignant liver neoplasms including gastrointestinal and neuroendocrine metastasis. The absolute contraindications to TACE include extensive liver disease and intractable infection [12]. Relative contraindications include portal vein thrombosis, uncorrectable coagulopathy, and poor renal function [12]. Those patients with tumor thrombus in the portal vein are considered contraindicated because of increased risk of liver failure due to hepatic ischemia [15]. Therefore, when selecting patients for TACE, those patients with advanced liver disease may neutralize the survival benefit of the intervention. Endovascular specialists should be aware that TACE is a reliable alternative for patients with unresectable hepatic neoplasms such as angiosarcoma.
